# Skin microbiome profile in people living with HIV/AIDS in Cameroon

**DOI:** 10.3389/fcimb.2023.1211899

**Published:** 2023-10-31

**Authors:** Kazuhiro Ogai, Benderli Christine Nana, Yukie Michelle Lloyd, John Paul Arios, Boonyanudh Jiyarom, Honore Awanakam, Livo Forgu Esemu, Aki Hori, Ayaka Matsuoka, Firzan Nainu, Rosette Megnekou, Rose Gana Fomban Leke, Gabriel Loni Ekali, Shigefumi Okamoto, Takayuki Kuraishi

**Affiliations:** ^1^ AI Hospital/Macro Signal Dynamics Research and Development Center (ai@ku), Institute of Medical, Pharmaceutical and Health Sciences, Kanazawa University, Kanazawa, Japan; ^2^ Department of Bio-engineering Nursing, Graduate School of Nursing, Ishikawa Prefectural Nursing University, Kahoku, Japan; ^3^ Faculty of Health Sciences, Institute of Medical, Pharmaceutical and Health Sciences, Kanazawa University, Kanazawa, Japan; ^4^ Biotechnology Center, University of Yaoundé I, Yaoundé, Cameroon; ^5^ Department of Animal Biology and Physiology of the Faculty of Science, University of Yaoundé I, Yaoundé, Cameroon; ^6^ Department of Tropical Medicine, Medical Microbiology and Pharmacology, John A. Burns School of Medicine, University of Hawaii at Manoa, Honolulu, HI, United States; ^7^ Institute of Medical Research and Medicinal Plant Studies, University of Yaoundé I, Yaoundé, Cameroon; ^8^ Faculty of Pharmacy, Institute of Medical, Pharmaceutical and Health Sciences, Kanazawa University, Kanazawa, Japan; ^9^ Department of Pharmacy, Faculty of Pharmacy, Hasanuddin University, Makassar, Indonesia; ^10^ Advanced Health Care Science Research Unit, Institute for Frontier Science Initiative, Kanazawa University, Kanazawa, Japan; ^11^ Division of Health Sciences, Graduate School of Medicine, Osaka University, Suita, Japan

**Keywords:** skin microbiome, AIDS, HIV, Cameroon, *Cutibacterium*

## Abstract

The presence of pathogens and the state of diseases, particularly skin diseases, may alter the composition of human skin microbiome. HIV infection has been reported to impair gut microbiome that leads to severe consequences. However, with cutaneous manifestations, that can be life-threatening, due to the opportunistic pathogens, little is known whether HIV infection might influence the skin microbiome and affect the skin homeostasis. This study catalogued the profile of skin microbiome of healthy Cameroonians, at three different skin sites, and compared them to the HIV-infected individuals. Taking advantage on the use of molecular assay coupled with next-generation sequencing, this study revealed that alpha-diversity of the skin microbiome was higher and beta-diversity was altered significantly in the HIV-infected Cameroonians than in the healthy ones. The relative abundance of skin microbes such as *Micrococcus* and *Kocuria* species was higher and *Cutibacterium* species was significantly lower in HIV-infected people, indicating an early change in the human skin microbiome in response to the HIV infection. This phenotypical shift was not related to the number of CD4 T cell count thus the cause remains to be identified. Overall, these data may offer an important lead on the role of skin microbiome in the determination of cutaneous disease state and the discovery of safe pharmacological preparations to treat microbial-related skin disorders.

## 1 Introduction

Skin is one of the most important innate immunological barriers available in humans. Together with the skin microbiome, this outer layer of protective elements have been known to play a vital role in a human’s life (Segre, 2006). In fact, impairment of the skin microbiome has been suggested to be closely associated with the occurrence of several dermatological conditions (Fredricks, 2001; Dethlefsen et al., 2007; Lunjani et al., 2019). Dysbiosis of the skin microbiome is involved in the occurrence of several dermatological disorders (Prescott et al., 2017) such as acne, atopic dermatitis, psoriasis, rosacea, and seborrheic dermatitis (Byrd et al., 2018). Recently, it has been reported that skin microbiota composition is subject to alteration in response to pharmacotherapeutic interventions e.g., the application of systemic antimicrobial drugs (Bayal et al., 2019). Thus, identifying factors that affect skin microbiota is crucial as they are important for maintaining skin homeostasis, thereby controlling human health.

Human immunodeficiency virus (HIV) infection can cause serious immune dysfunctions in the affected patients, which might modify the dynamic state of the human microbiome (Williams et al., 2016; D’Angelo et al., 2017). Over the years, HIV infection can progressively develop into acquired immune deficiency syndrome (AIDS) which leads to a more severe condition or even the death of the infected patients (Deeks et al., 2015). Almost a million people succumbed from AIDS each year, especially in developing countries, such as the ones located in Africa (Deeks et al., 2015; World Health Organization, 2022). In Cameroon, for example, HIV infection has been listed as the leading cause of all deaths and remains a threatening public health issue (Mbuagbaw et al., 2016). Of all HIV-related signatures, opportunistic dermatological infections such as prurigo, Kaposi’s sarcoma (KS), and other skin lesions are commonly observed in the AIDS patients (Deeks et al., 2015), implicating potential disturbances in the composition of skin microbiome. Although much focus has been given to study the skin-related disorders, research investigating the tripartite relationship between the immune-deficient status of the HIV-infected patient, the occurrence of dermatological conditions, and the composition of the skin microbiome remains underrepresented.

With the development of modern and advanced tools, human microbiome research has progressed exponentially (NIH Human Microbiome Portfolio Analysis Team, 2019). In relation to the alteration of microbiome under HIV-infected and/or AIDS-affected situations, several studies have been reported on gut (Dillon et al., 2016; Monaco et al., 2016), oral (Zhang et al., 2015; Ali et al., 2021; Li et al., 2021a), serum (Ali et al., 2021), and lung microbiome (Zhu et al., 2022). However, taking the topic of skin microbiome into account, to the best of our knowledge, no study has been reported on the skin microbiome in AIDS patients nor in HIV infected individuals without AIDS and how this may be associated to the presence of dermatological infections. In this study, we are seeking to characterize the composition of skin microbiota in HIV-infected patients, who may or may not have developed AIDS, to assess the presence of a dysbiosis due to immune suppression and how this dysbiosis is associated to the presence of skin conditions like prurigo or KS. Our findings shall provide important insights for further research to decipher the relationship between the patient’s skin microbiota and the development of opportunistic infections of the skin.

## 2 Materials and methods

### 2.1 Ethical considerations

This study was approved by the National Ethics Committee of Cameroon (approval no. 2018/06/1045/CE/CNERSH/SP), the hospital where the research was conducted, and the Medical Ethics Committee of Kanazawa University, where the next generation sequencing (NGS) was performed (approval no. 894). All research was performed in accordance with the Declaration of Helsinki. All participants were informed by a written document about the research, and written informed consent was obtained from all participants.

### 2.2 Study design and settings

This was a part of a cross-sectional study conducted in an HIV-clinic of the Efoulan District Hospital in Yaoundé, Cameroon, and in a single university in Japan. The inclusion criterion was being aged 21–65 years, because (1) the age of adulthood in Cameroon is 21 years old and thus it is deemed necessary to be 21 years old or older to understand and consent to this research, and (2) the skin microbiome of older people (older than ~65 years old) is reported to be affected by aging itself (Nagase et al., 2020) and thus several studies limit the age of participants being younger than or equal to 65 years old (Carrieri et al., 2021; Li et al., 2021b). The exclusion criteria were those who (1) had oral/topical antibiotics 1 week prior to the study, (2) were pregnant, or (3) were in a critical situation such as severe pneumonia, sepsis, pulmonary tuberculosis, toxoplasmosis, cryptococcosis, and meningoencephalitis. The participants were requested not to (1) take a bath or shower or (2) use emollients/creams on the sampling site, after midnight of the day of sampling (Nakatsuji et al., 2017), as these may alter the bacterial composition and interfere DNA extraction. Those who did not follow the instruction requested by the researchers were excluded.

### 2.3 Sample collection

Skin swabs were collected as described previously (Benderli et al., 2019; Ogai et al., 2022). In brief, Puritan HydraFlock Sterile Flocked Swab (25-3306-H; Puritan Medical Products Co., ME, USA) was presoaked in normal saline (S5815; Teknova, CA, USA) with 0.1% Tween 20 (28353-14; Nacalai Tesque, Inc., Kyoto, Japan) solution. Swabbing was then performed in a 5 × 5 cm^2^ on designated positions or in the entire area of KS. After swabbing, each swab head was cut and placed in a 1.5 mL microcentrifuge tube, carried in a cooler box with ice, and stored at -30°C until DNA extraction. To minimize sample degradation, DNA extraction was done in the same day of sampling.

### 2.4 DNA extraction

DNA from the swab head was performed as described previously (Ogai et al., 2018; Benderli et al., 2019; Ogai et al., 2022). Briefly, the swab heads were processed with the Kaneka Easy DNA Extraction Kit version 2 (KN-T110005; Kaneka Corp., Tokyo, Japan), followed by the enzymatic DNA extraction process with QIAamp DNA Mini Kit (51304; Qiagen N.V., Venlo, The Netherlands). The extracted DNA samples were stored at -30°C until NGS preparation.

### 2.5 NGS

The extracted DNA samples, along with the negative control (DNA extracted with swab head only) and positive control (DNA extracted from the ZymoBIOMICS Microbial Community Standard [D6300; Zymo Research Corp., Irvine, CA, USA]), were dedicated to the NGS analysis as described previously (Ogai et al., 2022). The same amount of the 16S rRNA gene from each sample was used to amplify the V3-V4 hypervariable region (Castelino et al., 2017; Zeeuwen et al., 2017; Ogai et al., 2018). After indexing with the Nextera XT Index Kit version 2 (FC-131-2001 to 2004; Illumina, Inc., San Diego, CA, USA), the library solution was loaded onto the MiSeq System (SY-410-1003; Illumina) with MiSeq Reagent Kit (version 3, 600 cycles; MS-102-3003; Illumina) and 15% PhiX Control (version 3; FC-110-3001; Illumina). The data from negative and positive controls are shown in **Supplementary Figure S1**.

### 2.6 Sequence analysis

The sequence analysis was done according to the Qiime2 instruction (Bolyen et al., 2019). Briefly, the raw fastq sequences were first quality-filtered and chimera-eliminated by DADA2 plugin (Callahan et al., 2016). Prior to analysis, the samples whose sequencing depth were <5,000 were discarded. For taxonomic classification of amplicon sequence variants (ASVs), q2-feature-classifier plugin (Bokulich et al., 2018) was used to construct the naïve Bayes classifier with the Silva database (version 138) (Quast et al., 2013). Beta diversity was calculated based on the Bray–Curtis distance followed by principal coordinate analysis by using q2-diversity plugin. Alpha diversity indices [observed ASVs, Faith’s phylogenetic diversity (PD), and Shannon index] were calculated by the q2-diversity plugin with the rarefaction at 5,000 depth of sequences.

### 2.7 Statistical analysis

All statistical analyses were performed using R statistical software (R Core Team, 2020) version 4.2.2. The data were expressed as means ± standard deviation or n (%) where appropriate. The boxplot denotes the 25th, 50th, and 75th percentile boxes with 25th percentile - 1.5 × IQR to 75th percentile + 1.5 × IQR whiskers. The relative abundance between the HIV-positive and HIV-negative population was compared by using the linear models for differential abundance analysis method (LinDA) (Zhou et al., 2022) with incorporating age and sex as covariates. For multiple comparisons of bacterial abundance, Benjamini-Hochberg’s false discovery rate control was employed. The significant differences in beta diversity between the two groups was assessed by a permutational analysis of variance with 9,999 permutations by adonis2 function of car package (Fox and Weisberg, 2018) of R software. Alpha diversity between the two groups were compared by using Mann–Whitney *U* test. For correlation analysis, Spearman’s correlation coefficient was used. *P*-values < 0.05 was considered statistically significant.

## 3 Results

### 3.1 16S rRNA-Based metagenomic analysis of skin microbes between the healthy and HIV-infected Cameroonians

This study first analyzed the relative abundance and prevalence of skin-resident microbes collected from healthy Cameroonians and the ones infected with HIV: Healthy (n = 26) and HIV-infected (n = 43). The demographic information is described in **Table 1**. Data of 16S rRNA gene sequencing analysis of skin swabs from healthy Cameroonians were overlapped with our previous study (Ogai et al., 2022). However, we applied ASV-based analysis to compare microbiome difference in higher resolution.

**Table 1:** Demographic data.

	HIV^-^ (*n* = 26)	HIV^+^ (*n* = 43)
Sex, female, *n* (%)	20 (76.9)	34 (79.1)
Age, y, mean ± SD	31.2 ± 8.4	40.0 ± 9.3
BMI, mean ± SD	26.4 ± 4.1	25.4 ± 6.3
Time since HIV diagnosis, month, mean (min – max)	–	56.7 (12 – 200)
CD4 count, cells/μL, mean ± SD	–	456.4 ± 301.5
HIV medication (ART), *n* (%)	–	41 (95.3)
Time on ART, month, mean (min – max)	–	24.8 (0 – 96)
ART Regimen, *n* (%) TDF+3TC+EFV Others Unknown	- - -	33 (76.7) 7 (16.3) 3 (7.0)

BMI, body mass index; CD4, cluster of differentiation 4; HIV, human immunodeficiency virus; ART, antiretroviral therapy; TDF, Tenofovir disoproxil fumarate; 3TC, Lamivudine; EFV, efavirenz.

**Figure 1:**
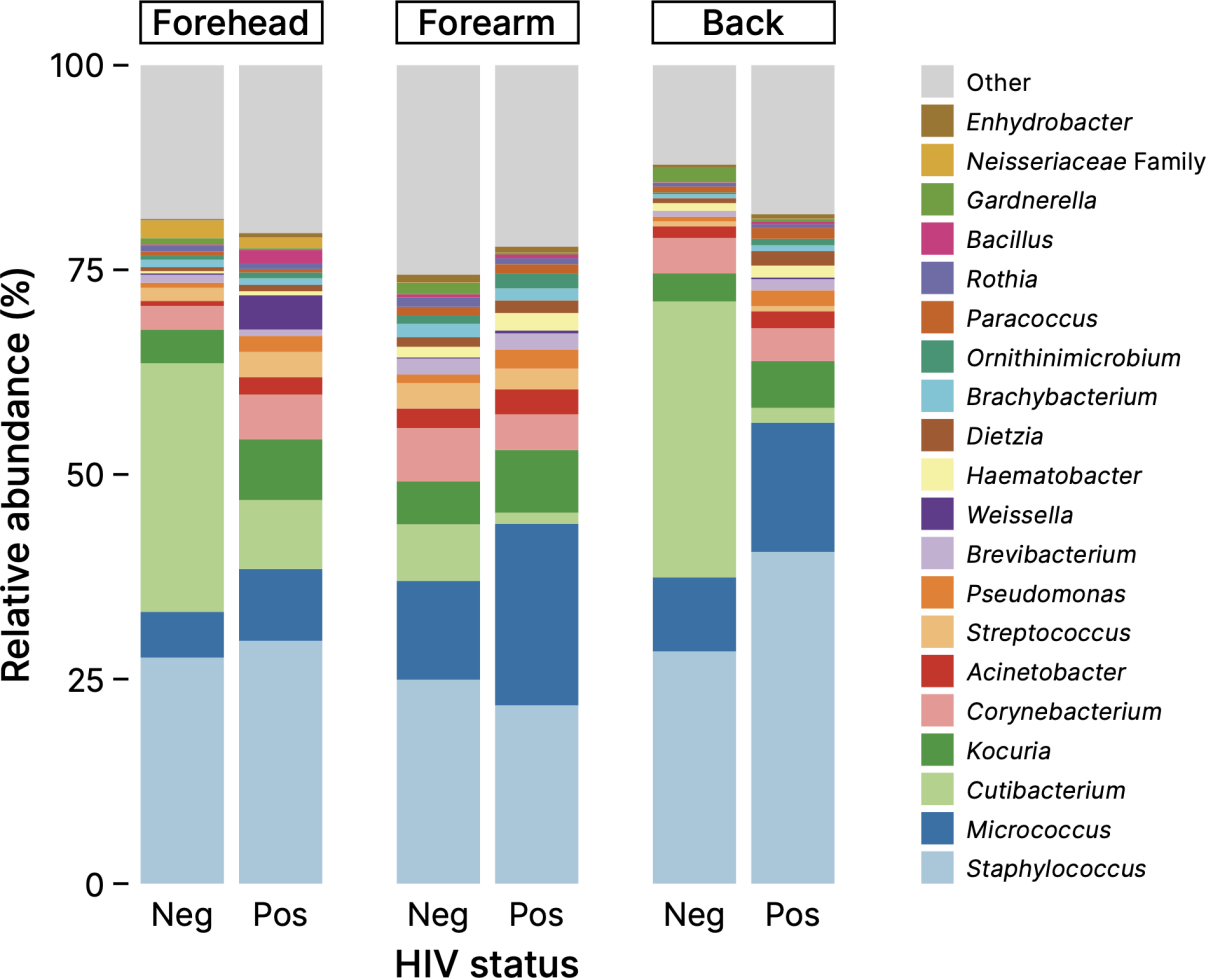
Relative abundance of skin microbiome (top 20). Neg, negative; pos, positive.

**Figure 1** shows a comparison of the skin microbiome compositions between healthy Cameroonians and their HIV-infected counterparts in the three skin sites: forehead, right forearm, and the mid-upper back. The composition of skin microbiome of all samples are shown in **Supplementary Figure S2**. The microbiome of the Cameroonian skin samples, in the presence or absence of HIV infection, can be classified into more than 20 genera that primarily belong to three phyla: Actinobacteria, Firmicutes, and Proteobacteria. At the genus level, most obtained sequences were *Staphylococcus*, *Cutibacterium*, *Micrococcus*, *Corynebacterium*, *Kocuria*, and, to a lesser extent, *Acinetobacter* and *Streptococcus* (**Figure 1**). In the samples collected from the surface of KS, the main bacterial genera were common, but their proportions were more diverse (**Supplementary Figure S3**).

**Figure 2:**
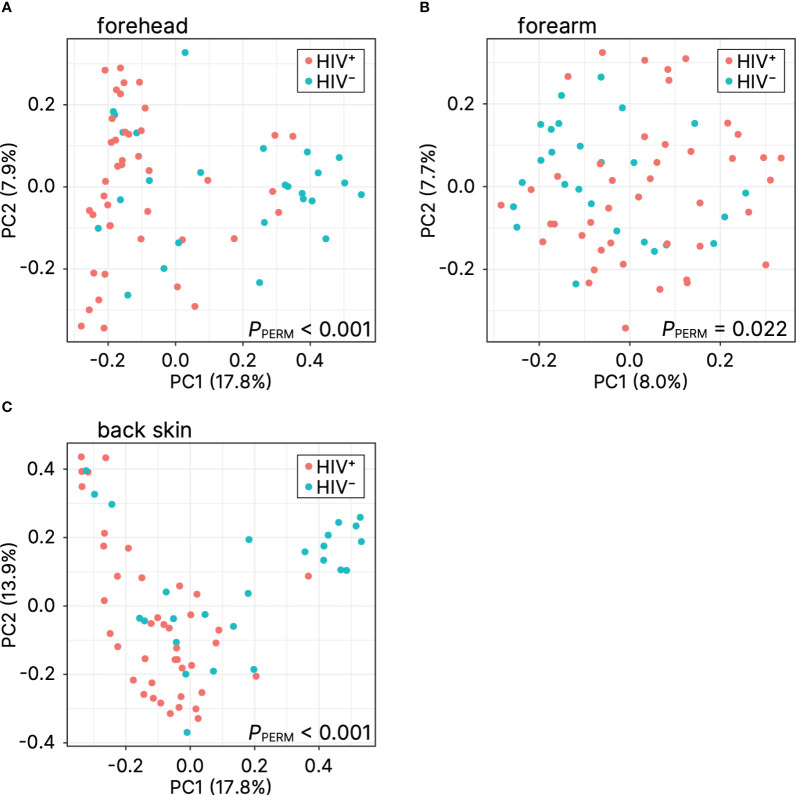
Principal coordinate analysis of beta diversity (Bray–Curtis distance) for **(A)** forehead, **(B)** forearm, and **(C)** back skin. PC, principal coordinate; *P*_PERM_, *P*-value of the permutational analysis of variance.

The microbial differences between the HIV-positive and HIV-negative participants were further confirmed by the beta diversity analysis using the Bray–Curtis distance. The principal component analysis of beta diversity showed that the skin samples (forehead, forearm, and back skin) obtained from HIV-infected Cameroonian constituted significantly different clusters from healthy Cameroonian participants (**Figure 2**). This result suggests that there is a difference of microbiome profiles between healthy and HIV-infected people.

**Figure 3:**
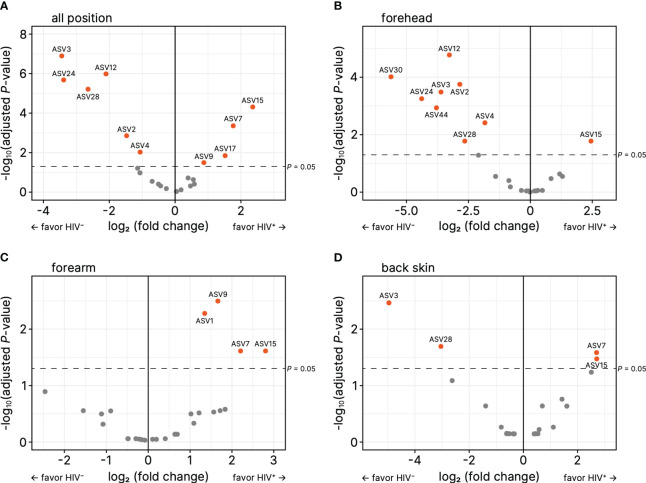
Volcano plots for relative abundance of amplicon sequence variances (ASVs) on **(A)** forehead, **(B)** forearm, and **(C)** back skin. The horizontal axes denote the logarithm of relative fold change (based on HIV-negative) to base 2, and the vertical axes denote the negative of logarithm of adjusted *P*-values to base 10. The ASVs that have *P* < 0.05 were orange colored.

ASV-based analysis showed that the genus or species of *Micrococcus* (ASV1, ASV9), *Kocuria marina* (ASV7), *Pseudomonas* (ASV15), *Staphylococcus* (ASV17) were significantly higher in all or some parts of skin sites of the HIV-infected Cameroonians in comparison to their healthy counterparts (**Figure 3**; **Table 2**). In contrast, the genus or species of *Cutibacterium* (ASV3), *Staphylococcus* (ASV2, ASV4, ASV30), *Corynebacterium tuberculostearicum* (ASV12), *Corynebacterium* (ASV28), *Delftia* (ASV24), *Stenotrophomonas* (ASV44) were significantly lower in the HIV-infected Cameroonians (**Figure 3**; **Table 3**).

**Table 2:** Amplicon sequence variances (ASVs) that showed significant increase in HIV^+^ population.

Increase in HIV^+^ population					
ASV ID	Genus (+species)	All position	Forehead	Forearm	Back skin
ASV1	*Micrococcus*			↑	
ASV7	*Kocuria marina*	↑		↑	↑
ASV9	*Micrococcus*	↑		↑	
ASV15	*Pseudomonas*	↑	↑	↑	↑
ASV17	*Staphylococcus*	↑			

Only ASVs that passed (1) prevalence > 50% and (2) abundance > 0.25% were analyzed.

In the order of mean abundance.

Significancy was tested by a linear regression framework for differential abundance analysis (LinDA) with adjusted P-values by Benjamini-Hochberg’s false discovery rate control.

All linear regression tests were adjusted for age and sex.

“All position” means the position term was incorporated into the covariance term of the linear regression test.

↑, significant increase. Raw statistics are shown in **Supplementary Table S1**.

**Table 3:** Amplicon sequence variances (ASVs) that showed significant decrease in HIV^+^ population.

Decrease in HIV^+^ population					
ASV ID	Genus (+species)	All position	Forehead	Forearm	Back skin
ASV3	*Cutibacterium*	↓	↓		↓
ASV4	*Staphylococcus*	↓	↓		
ASV2	*Staphylococcus*	↓	↓		
ASV12	*Corynebacterium* *tuberculostearicum*	↓	↓		
ASV30	*Staphylococcus*		↓		
ASV24	*Delftia*	↓	↓		
ASV44	*Stenotrophomonas*		↓		
ASV28	*Corynebacterium*	↓	↓		↓

Only ASVs that passed (1) prevalence > 50% and (2) abundance > 0.25% were analyzed.

In the order of mean abundance.

Significancy was tested by a linear regression framework for differential abundance analysis (LinDA) with adjusted P-values by Benjamini-Hochberg’s false discovery rate control.

All linear regression test was adjusted for age and sex.

“All position” means the position term was incorporated into the covariance term of the linear regression test.

↓, significant decrease. Raw statistics are shown in **Supplementary Table S1**.

Overall, these findings indicated that HIV infection does affect the composition of skin microbiome in the Cameroonians.

### 3.2 Higher alpha diversity in HIV-infected Cameroonians

**Figure 4:**
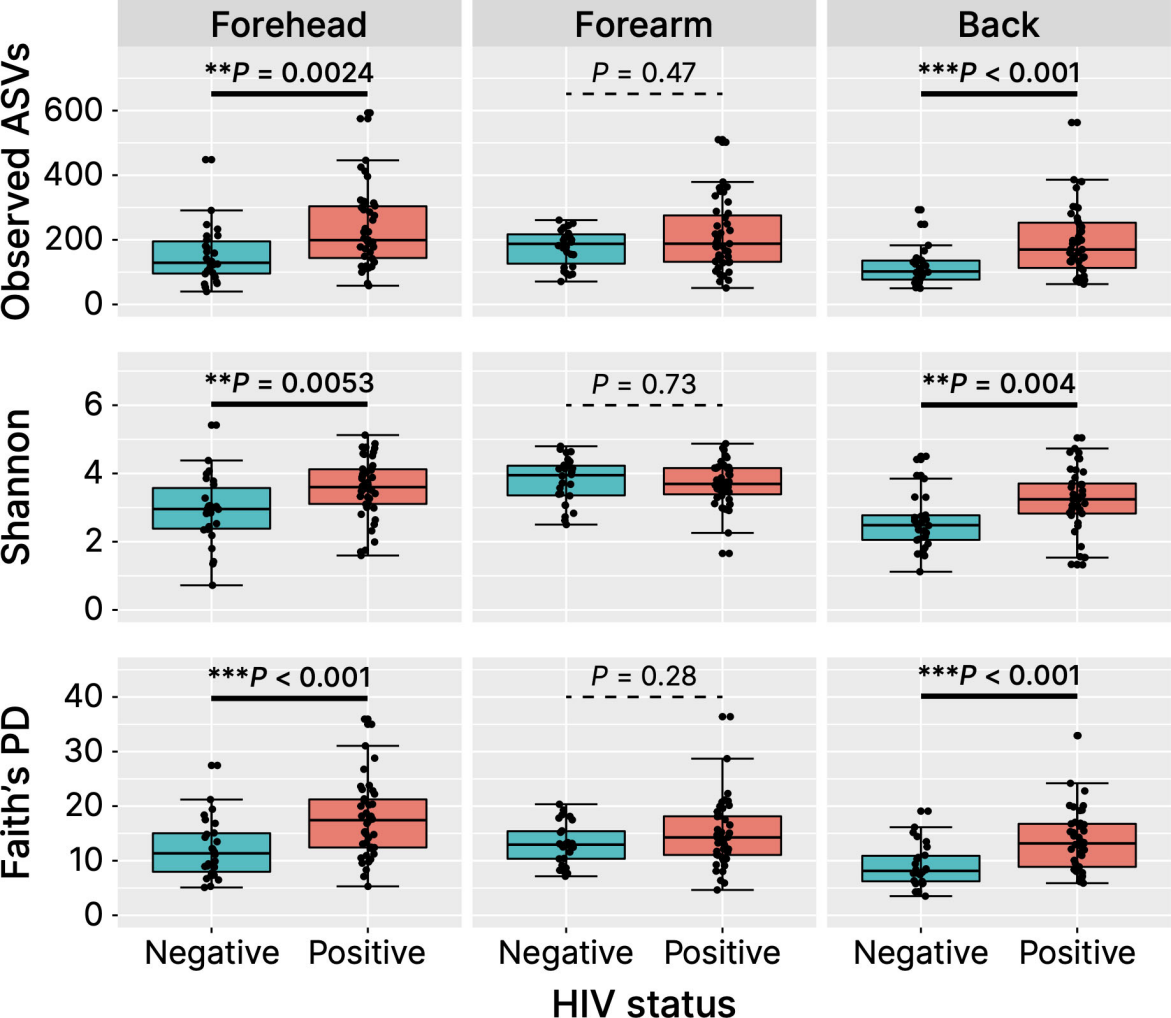
Alpha diversity metrices. ASVs, amplicon sequence variances; PD, phylogenetic diversity. **P < 0.01, ***P < 0.001.

We calculated the alpha diversity metrics of the skin microbiome between healthy Cameroonians and the HIV-infected individuals in the forehead, forearm and the back skin, namely the number of observed ASVs, Faith’s PD, and Shannon index (**Figure 4**). HIV-infected Cameroonians have a much diverse skin-resident flora at the forehead and the back, with a significantly higher alpha diversity metrices. However, no significant difference in the skin microbiome diversity of the forearm skin between both groups. These findings suggest that HIV-infected people have more diverse bacterial species than their healthy counterparts in some parts of their skin.

### 3.3 Irrelevant correlation of CD4+ T cells count with bacterial species abundance

The close correlation of CD4 T cells count with the progressiveness of HIV status has been widely suggested (Deeks et al., 2015; Kestens and Mandy, 2017). To investigate whether the number of CD4 T cells is likely correlated with the discrepancies of skin microbe composition observed in this study, an experiment to determine the CD4 T cell count was carried out. As revealed in **Table 4**, we observed that there is no correlation between the number of CD4 T cell count with the abundance of bacterial species recovered from the skin samples of HIV-infected Cameroonians.

**Table 4:** Spearman’s correlation coefficients between the CD4^+^ cell number and the relative abundance of each amplicon sequence variance (ASV).

ASV ID	Forehead	Forearm	Back skin
ASV3 (*Cutibacterium* sp.)	-0.32	—	—
ASV7 (*Kocuria marina*)	-0.13	—	—
ASV22 (*Weissella* sp.)	-0.03	—	—
ASV25 (*Acinetobacter_haemolyticus*)	0.24	0.38	—

Only significant (P < 0.05) correlation coefficients are displayed: no significant correlation is denoted as —.

## 4 Discussion

In this study, we carried out comprehensive analysis of the skin microbiome profile of healthy Cameroonians, at three different skin sites: forehead, right forearm, and the mid-upper back, and compared them to the ones infected with HIV. Taking advantage of the NGS method and ASV-based bioinformatic analysis, we assessed the dynamic variations of Cameroonians skin microbiome and ascertain whether topological elements might play distinctive roles in the skin microbiome diversity in the event of HIV infection. Our ASV-based analysis indicated that, in HIV-infected Cameroonians, the relative number of some bacteria increased and some decreased. Furthermore, our results clearly showed that the alpha-diversity of the skin microbiome was greater, and beta-diversity was distinct from healthy counterparts. These findings collectively suggest that HIV infection affects human skin microbiome.

To inspect the compositional difference in detail, we observed that the relative abundance of *Cutibacterium* species (spp.) was significantly lower in the HIV-infected Cameroonians than their healthy counterparts. In our previous study, we confirmed that *Cutibacterium* spp. was less observed in the skin of healthy Cameroonians than in that of healthy Japanese people (Ogai et al., 2022), probably due to the less sebum-rich ecological nature of Cameroonians skin that might support *Cutibacterium* spp. growth. It is important to note that the role of *Cutibacterium* spp. in the cutaneous homeostasis and skin health has been reported (Bolla et al., 2020; Claesen et al., 2020; Rozas et al., 2021). Hence, although the cause of *Cutibacterium* spp. decline remains to be identified, this finding is particularly interesting because HIV infection may further reduce the already low levels of *Cutibacterium* spp., which may adversely affect to the skin homeostasis and lead to undesirable phenotypes observed during the state of disease.

It is widely known that the skin microbiome can be modified by the skin-related diseases such as atopic dermatitis and psoriasis. For example, higher *Staphylococcus aureus* and *Corynebacterium* is reported in atopic dermatitis (Kobayashi et al., 2015), and colonization of *Staphylococcus*, *Streptococcus*, *Finegoldia*, and *Corynebacterium* is reported to be involved in psoriasis (Hsu et al., 2020). On the other hand, changes in skin microbiome of HIV^+^ patients (**Figure 1**; **Tables 2**, **3**) are different from those found in major skin diseases, highlighting the uniqueness of skin microbiome under HIV infection. However, there are emerging interest in skin “mycobiome” in recent years, and it is reported that *Malassezia* and *Candida* fungi are involved in psoriasis (Gupta et al., 2022). Analysing fungal composition, or mycobiome, of HIV patients’ skin might also give new insights about HIV-related changes in skin microorganisms.

The composition of skin microbiome can be affected by several intrinsic factors, such as aging, sex, and racial differences (Wilantho et al., 2017; Khmaladze et al., 2020; Boxberger et al., 2021; Kim et al., 2021). Additionally, extrinsic factors such as hygiene, lifestyle, climate and/or geographical differences are also stated to be accountable for variations in the skin microbiome profile (Lehtimaki et al., 2017; Khmaladze et al., 2020; Boxberger et al., 2021). We previously showed that Cameroonian people have significantly higher alpha diversity in skin microbiome than Japanese people do, which has been attributed to both intrinsic and extrinsic factors (Ogai et al., 2022). In this study, we observed that the alpha diversity was significantly higher in the HIV-positive individuals. Given that the participants in this study resided in the close region (Yaoundé city) of the country, and sex and age are not so different (young- to middle- aged), it is speculated that HIV infection itself, other than environmental and innate factors, influences the diversity of the skin microbiota. Further studies would be desired whether increased alpha-diversity indicates the likelihood of opportunistic skin infection. Moreover, the cause for such differences shall be an interesting venue for future research. Is it the result of HIV infection? How might HIV infection alter the number of bacterial species? Since we found that there is no correlation between the number of CD4 T cell count with the abundance of bacterial species recovered from the skin samples of HIV-infected Cameroonians (**Table 4**), we consider that difference in the cellular adaptive immune status, at least the CD4 T cells, might not the primary cause for the different skin microbiome.

Another important question is whether the observed skin microbiome distinction alters the landscape of skin homeostasis in HIV-infected people, such as the integrity of skin structure and skin opportunistic infections. Such information might provide insights on how skin microbiome modulates its physiology and how those interactions may influence the patients state during the course of HIV infection. This, in the end, may provide important leads in the discovery of effective pharmacological preparations to manage microbial-related disorders in the skin.

Through this study, we could obtain the changes of skin microbiome in HIV patients. Although we need further studies to look into the relationship between the microbial changes and skin condition and immunological status, information of skin microbiome could be used for predicting and preventing the opportunistic infection of the skin in HIV patients.

## 5 Conclusion

In this study, we have shown the distinct skin microbiome between HIV-positive and HIV-negative Cameroonian people. This study may provide a brief but fundamental information to better understand skin symptoms and crosstalk between skin and body via the skin microbiome under the influence of HIV infection.

## Data availability statement

The datasets presented in this study can be found in online repositories. The names of the repository/repositories and accession number(s) can be found below: https://www.ddbj.nig.ac.jp/, DRA011596 and DRA016187.

## Ethics statement

The studies involving humans were approved by National Ethics Committee of Cameroon (approval no. 2018/06/1045/CE/CNERSH/SP) Medical Ethics Committee of Kanazawa University (approval no. 894). The studies were conducted in accordance with the local legislation and institutional requirements. The participants provided their written informed consent to participate in this study.

## Author contributions

Conceptualization: GE, SO, and TK. Methodology: KO, YL, AH, AM, SO, and TK. Software: KO. Validation: KO, BN, AM, FN, and TK. Formal analysis: KO, BN, and AM. Investigation: KO, BN, YL, JA, BJ, HA, LE, AH, AM, GL, and TK. Resources: BN, HA, LE, RM, RL, and GL. Data curation: KO, AM, FN, and TK. Writing—original draft preparation: KO, FN, and TK. Writing—review and editing: KO, FN, and TK. Visualization: KO and AM. Supervision: RM, RL, GL, SO, and TK. Project administration: GL, SO, and TK. Funding acquisition: KO, YL, GL, SO, and TK. All authors have read and agreed to the published version of the manuscript.
