# Supplementary material for: Skin microbiome profile in people living with HIV/AIDS in Cameroon
Source: Front Cell Infect Microbiol. 2023 Oct 31;13:1211899. doi: 10.3389/fcimb.2023.1211899 (PMC10644231; doi:10.3389/fcimb.2023.1211899)
Supplement: Supplementary file 1 [file DataSheet_1.docx]

Supplementary Material

Skin microbiome profile in people living with HIV/AIDS

**Kazuhiro Ogai^1, 2, 8^, Benderli Christine Nana^3, 4^, Yukie Michelle Lloyd^5^, John Paul Arios^5^, Boonyanudh Jiyarom^5^, Honore Awanakam^3^, Livo Forgu Esemu^3, 6^, Aki Hori^7^, Ayaka Matsuoka^8^, Firzan Nainu^7, 9^, Rosette Megnekou^3, 4^, Rose Gana Fomban Leke^3, 6^, Gabriel Loni Ekali^3, *^, Shigefumi Okamoto^8, 10, 11*^ and Takayuki Kuraishi^7, *^**

*** Correspondence:**

Gabriel Loni Ekali: [eloni2000@yahoo.com](mailto:eloni2000@yahoo.com)

Shigefumi Okamoto: [sokamoto@sahs.med.osaka-u.ac.jp](mailto:sokamoto@sahs.med.osaka-u.ac.jp)

Takayuki Kuraishi: [tkuraishi@staff.kanazawa-u.ac.jp](mailto:tkuraishi@staff.kanazawa-u.ac.jp)

# Supplementary Figure

**Supplementary Figure S1**. Negative (A) and positive (B) experimental controls. The negative control was prepared by processing a swab without skin swabbing, and the positive control was derived from ZymoBIOMICS Microbial Community Standard (D6300; Zymo Research Corp., Irvine, CA, USA), both were processed along with the other skin swabs.

These data are from the same sequence run used in this study and the previous study (Ogai et al., 2022), and thus the data shown here is identical to that in Supplementary Figure S3 of the said study (https://static-content.springer.com/esm/art%3A10.1038%2Fs41598-022-05244-5/MediaObjects/41598_2022_5244_MOESM1_ESM.pdf).

**Supplementary Figure S2.** Relative abundances of skin microbiome of all analyzed data.


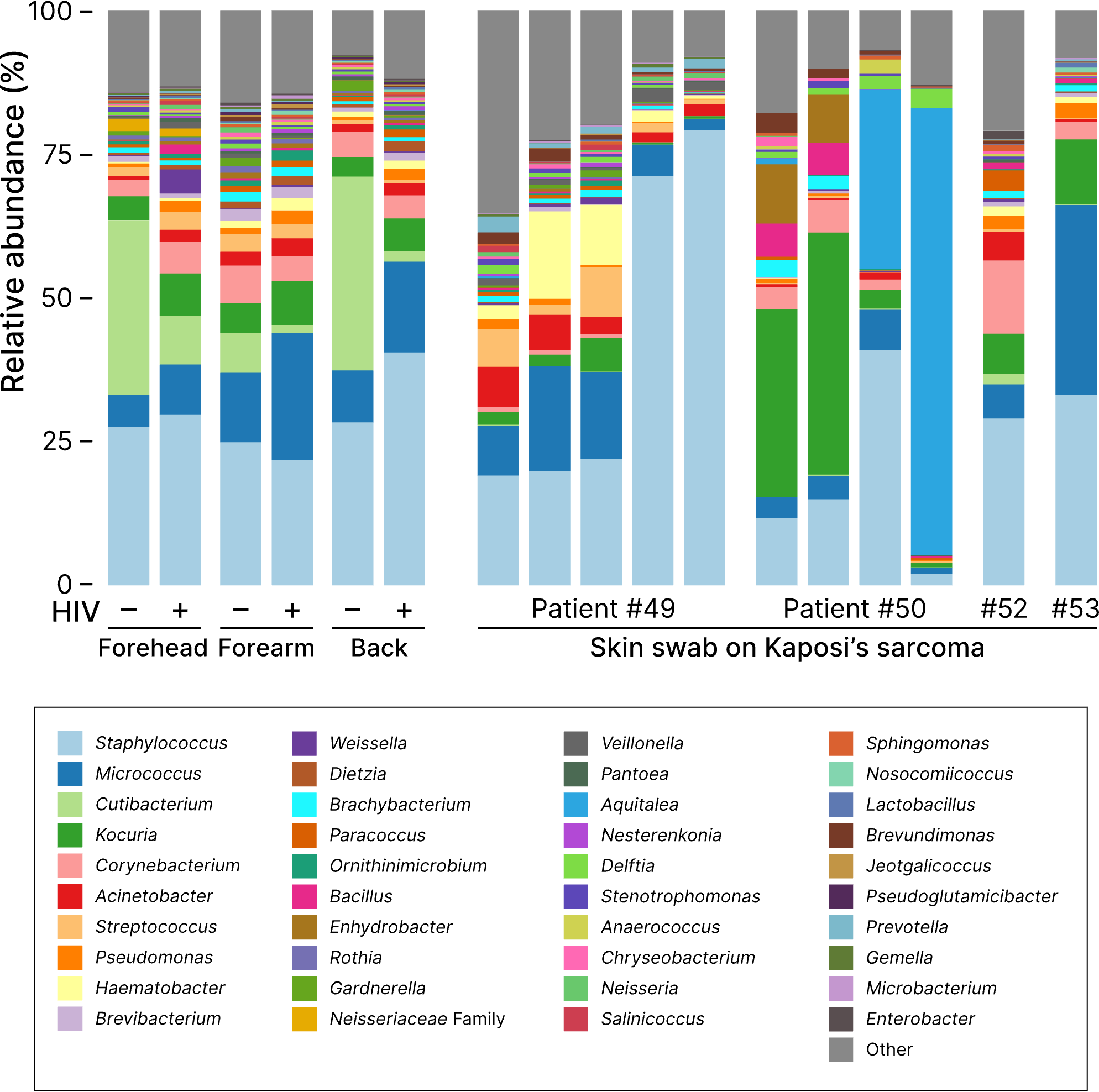


**Supplementary Figure S3.** Relative abundances of skin microbiome on Kaposi’s sarcoma.

# Supplementary Table

**Supplementary Table S1.** Results of the linear models for differential abundance analysis (LinDA) in each location and overall change.

- Attached as a separate file.

**References**

Ogai, K., Nana, B.C., Lloyd, Y.M., Arios, J.P., Jiyarom, B., Awanakam, H., et al. (2022). Skin microbiome profile of healthy Cameroonians and Japanese. Sci. Rep. 12, 1364. doi: 10.1038/s41598-022-05244-5.
